# Evaluation of Ortho VITROS and Roche Elecsys S and NC Immunoassays for SARS-CoV-2 Serosurveillance Applications

**DOI:** 10.1128/spectrum.03234-22

**Published:** 2023-06-22

**Authors:** Hasan Sulaeman, Eduard Grebe, Honey Dave, Lily McCann, Clara Di Germanio, Aditi Sanghavi, Victoria Sclar, Daniel W. Bougie, Garrick Chatelain, Brad J. Biggerstaff, Jefferson M. Jones, Natalie J. Thornburg, Steve Kleinman, Mars Stone, Michael P. Busch

**Affiliations:** a Vitalant Research Institute, San Francisco, California, USA; b SACEMA, Stellenbosch University, Stellenbosch, South Africa; c Versiti Blood Center, Milwaukee, Wisconsin, USA; d The Blood Center, New Orleans, Louisiana, USA; e Centers for Disease Control and Prevention, Fort Collins, Colorado, USA; f COVID-19 Response Team, Centers for Disease Control and Prevention, Atlanta, Georgia, USA; g University of British Columbia, Victoria, British Columbia, Canada; h Department of Laboratory Medicine, University of California, San Francisco, California, USA; National Chung Hsing University

**Keywords:** COVID-19, SARS-CoV-2, antibodies, serology, serosurveillance, serosurvey

## Abstract

SARS-CoV-2 seroprevalence studies are instrumental in monitoring epidemic activity and require well-characterized, high-throughput assays, and appropriate testing algorithms. The U.S. Nationwide Blood Donor Seroprevalence Study performed monthly cross-sectional serological testing from July 2020 to December 2021, implementing evolving testing algorithms in response to changes in pandemic activity. With high vaccine uptake, anti-Spike (S) reactivity rates reached >80% by May 2021, and the study pivoted from reflex Roche anti-nucleocapsid (NC) testing of Ortho S-reactive specimens to parallel Ortho S/NC testing. We evaluated the performance of the Ortho NC assay as a replacement for the Roche NC assay and compared performance of parallel S/NC testing on both platforms. Qualitative and quantitative agreement of Ortho NC with Roche NC assays was evaluated on preselected S/NC concordant and discordant specimens. All 190 Ortho S+/Roche NC+ specimens were reactive on the Ortho NC assay; 34% of 367 Ortho S+/Roche NC- specimens collected prior to vaccine availability and 43% of 37 Ortho S-/Roche NC+ specimens were reactive on the Ortho NC assay. Performance of parallel S/NC testing using Ortho and Roche platforms was evaluated on 200 specimens collected in 2019 and 3,903 study specimens collected in 2021. All 200 pre-COVID-19 specimens tested negative on the four assays. Cross-platform agreement between Roche and Ortho platforms was 96.4% (3,769/3,903); most discordant results had reactivity close to the cutoffs on the alternate assays. These findings, and higher efficiency and throughput, support the use of parallel S/NC testing on either Roche or Ortho platforms for large serosurveillance studies.

**IMPORTANCE** Seroprevalence studies like the U.S. Nationwide Blood Donor Seroprevalence Study (NBDS) have been critical in monitoring SARS-CoV-2 epidemic activity. These studies rely on serological assays to detect antibodies indicating prior infection. It is critical that the assays and testing algorithms used in seroprevalence studies have adequate performance (high sensitivity, high specificity, ability to discriminate vaccine-induced and infection-induced antibodies, etc.), as well as appropriate characteristics to support large-scale studies, such as high throughput and low cost. In this study we evaluated the performance of Ortho’s anti-nucleocapsid assay as a replacement for the Roche anti-nucleocapsid assay and compared performance of parallel anti-spike and anti-nucleocapsid testing on both platforms. These data demonstrate similar performance of the Ortho and Roche anti-nucleocapsid assays and that parallel anti-spike and anti-nucleocapsid testing on either platform could be used for serosurveillance applications.

## INTRODUCTION

Shortly after SARS-CoV-2 was identified as a possible pandemic by the WHO, serosurveillance programs were launched in many countries to monitor infection and vaccination rates for the emerging pathogen. Timely estimates of seroprevalence in a population, reflective of cumulative infection incidence or vaccinations, are important in pandemic surveillance and are used to inform policies, including the implementation and assessment of impact of interventions (1–3).

In choosing assays for large-scale serosurveillance studies, assay performance characteristics, testing algorithms, and operational feasibility are important considerations. Many serological assays for anti-SARS-CoV-2 antibodies have been used in serosurveillance studies globally, with various performance characteristics (4–6). Some studies utilize lower throughput in-house assays such as ELISAs (7, 8), while other studies utilize commercial higher throughput assays from major manufacturers such as Abbott, Roche, Euroimmun, Wantai, and Ortho Clinical Diagnostics (5, 9, 10). For optimal selection and use of assays in serosurveillance, three performance characteristics are key: sensitivity, specificity, and durability of antibody detection (11). While most commercially available SARS-CoV-2 serological assays have good specificity and sensitivity in recently infected individuals, durability of antibody detection varies considerably (11, 12). Moreover, for larger serosurveillance programs, operational feasibility considerations, such as high-throughput testing on fully automated platforms in multiple laboratories and cost, are important considerations in ensuring timely generation and reporting of accurate results.

The United States Centers for Disease Control and Prevention (CDC), in collaboration with Vitalant Research Institute and 17 U.S. blood collection organizations, launched the Nationwide Blood Donor Seroprevalence (NBDS) Study in July 2020 (13, 14) to monitor rates of SARS-CoV-2 seroreactivity in 66 regions, representing all 50 states, Washington, D.C. and Puerto Rico. The NBDS program included an evaluation of performance characteristics of 21 commercially available, high-throughput serological assays (11), which was executed in part to identify appropriate assays and algorithms for the NBDS study. As the pandemic evolved and vaccination of the U.S. donor population expanded, the study testing algorithm had to be modified. The initial testing algorithm screened donor specimens using the Ortho VITROS anti-SARS-CoV-2 Spike Total Ig assay (Ortho S), reflexing reactive specimens for testing on the Roche Elecsys NC anti-SARS-CoV-2 nucleocapsid assay (Roche NC). In early 2021, S-based vaccine uptake in the United States increased rapidly, resulting in anti-S reactivity rates of 80% by May 2021, which made the reflex testing algorithm impractical. Consequently, in mid-2021 the NBDS program evaluated and then implemented parallel anti-S and anti-NC testing on the Ortho platform, providing substantial logistical advantages and lower cost of testing. Continued anti-S testing, in the parallel testing algorithm, allowed for observation of seroreactivity induced by vaccination alone. For parallel S/NC testing performed on the same platform, both assays should have acceptable performance characteristics for serosurveillance, including excellent sensitivity, specificity, and durability in antibody detection for >1 year after infection or vaccination. To validate the Ortho NC assay, we first evaluated the performance of the Ortho NC assay relative to the Roche NC assay on selected archived samples from the NBDS Study. We also conducted a comparative assessment of single-platform Ortho and Roche S/NC parallel testing to support use of either of these platforms in serosurveillance studies and to allow for comparison of findings from studies conducted using the Roche S/NC assays to results of the NBDS study using the Ortho S/NC assays.

## RESULTS

### Comparative assessment of Roche and Ortho NC SARS-CoV-2 immunoassays.

**FIG 1:**
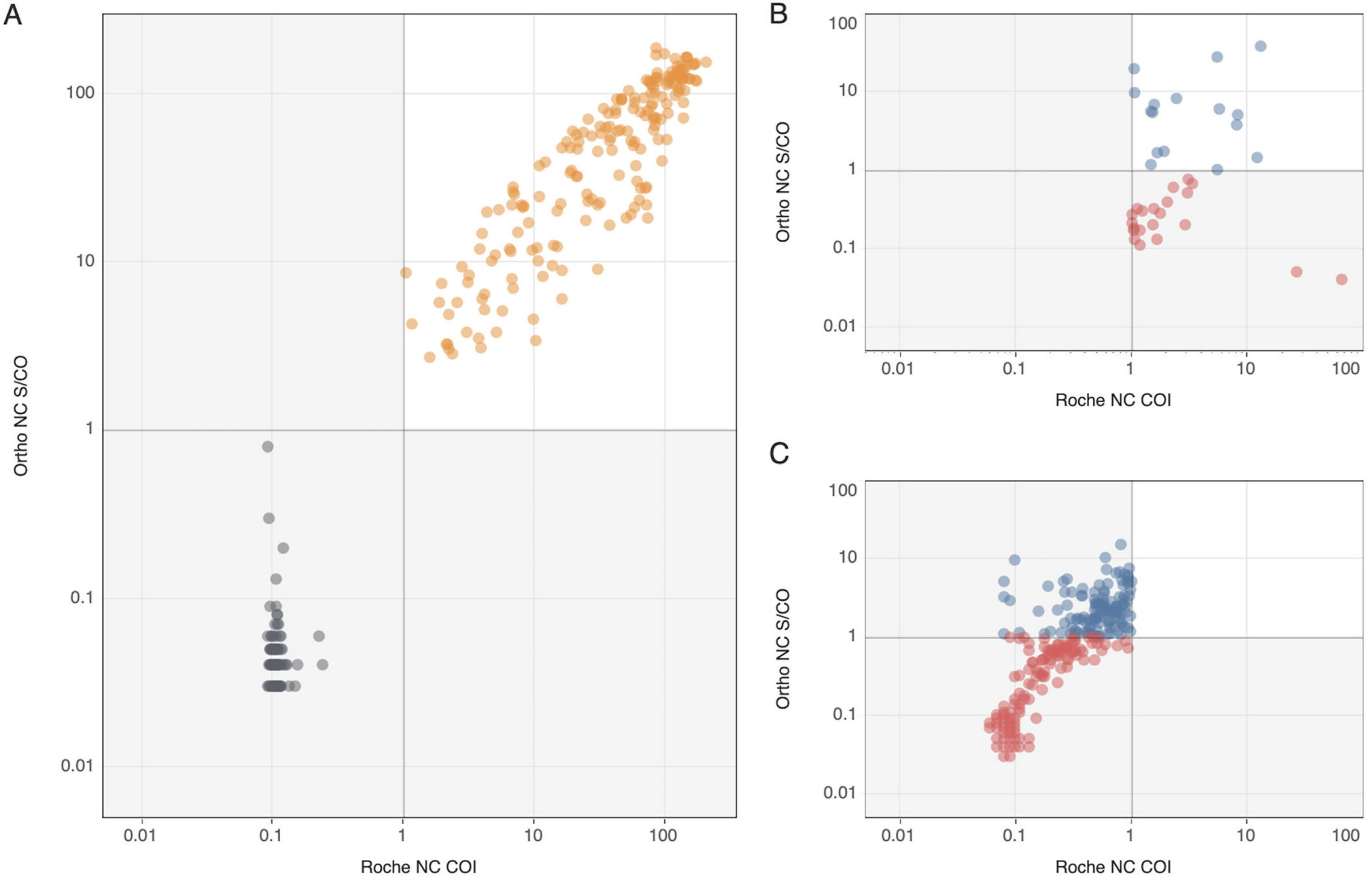
Comparison of Ortho and Roche nucleocapsid (NC) assays numeric outputs (S/CO and COI). Ortho NC and Roche NC numeric outputs on preselected specimens tested to validate the Ortho NC assay to replace the Roche NC assay. A) Ortho S+/Roche NC+ shown in orange and Ortho S-/Roche NC- shown in black (*n* = 190 and *n* = 200;, respectively). B) Ortho S-/Roche NC+ (*n* = 37). C) Ortho S+/Roche NC- (*n* = 367). In (B) and (C), red and blue symbols denote Ortho NC nonreactive and reactive specimens, respectively. Gray lines on the axes denote manufacturer-set cutoffs.

Both Roche NC and Ortho NC assays tested nonreactive on all 200 specimens collected prior to the pandemic in 2019 (100% specificity, 95% CI: 98.5 to 100%) and both assays were reactive on all 190 selected Ortho S+/Roche NC+ specimens tested as part of the NDBS Study. Fig. 1A shows the numeric outputs of the Roche NC and Ortho NC assays for preselected Ortho S+/Roche NC+ specimens that were tested as part of the NBDS Study and Ortho S-/Roche NC- specimens collected during 2019. For the concordant Ortho S+/Roche NC+ reactive specimens, the Ortho NC signal to cutoff ratio (S/CO) and the Roche NC cutoff index (COI) were highly correlated (Spearman’s correlation coefficient of 0.89; *P* < 0.001).

Of 367 Ortho S+/Roche NC- specimens donated in 2020 prior to vaccine availability, 124 (34%) tested reactive on the Ortho NC assay. Fig. 1B shows the distribution of reactivity in the NC assays and demonstrates the relatively low signal intensity for both low-level positive and gray zone negative specimens compared to concordant S+/NC+ specimens shown in panel A, right upper quadrant. Of the 37 Ortho S-/Roche NC+ specimens identified from the NBDS Study sites that performed systematic Roche NC screening of all donations, 16 (43%) tested reactive on the Ortho NC assay (Fig. 1B).

Among the Ortho S+/Roche NC+ specimens (Fig. 1A), 10 (5%) had a signal intensity between 0.5 and 5 on both the Ortho NC and Roche NC assays; none of the 200 Ortho S-/Roche S- specimens had gray zone reactivity. In contrast, among the 404 Ortho S-/Roche NC+ specimens (Fig. 1B) and Ortho S+/Roche NC- (Fig. 1C) specimens, 80 (20%) had signal intensities between 0.5 and 5 on both the Ortho NC and Roche NC assays.

### Evaluation of Roche and Ortho platforms for parallel S/NC testing.

**FIG 2:**
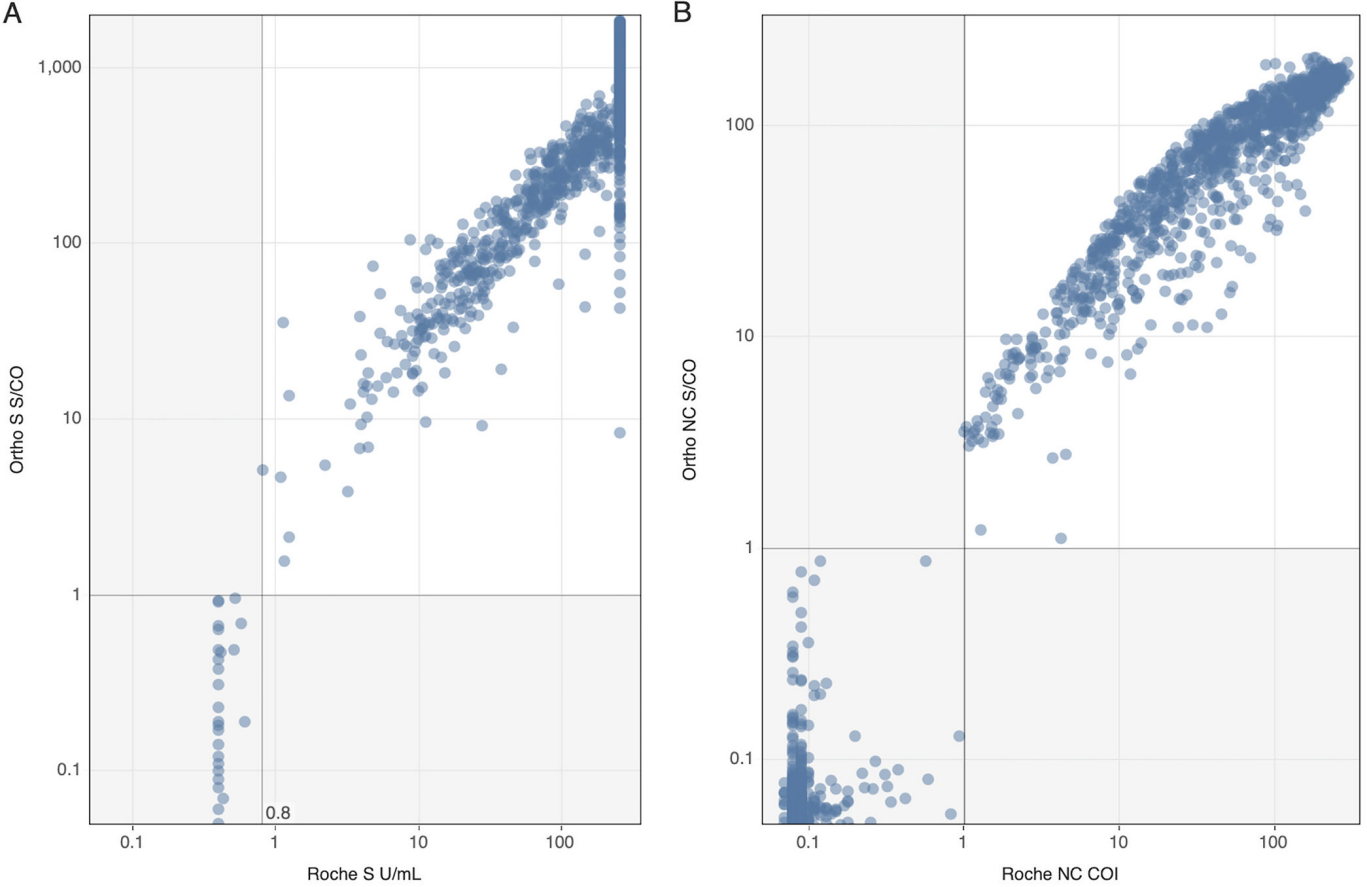
Numeric outputs from Ortho and Roche parallel testing for the antigen concordant nonreactive and reactive groups (S-/NC- and S+/NC+; *n* = 2365). (A) Numeric outputs of the two S assays. Gray line at 0.8 and 1 denote manufacturer set cutoff for the Roche and Ortho S assays, respectively. (B) Numeric outputs of the two NC assays. Gray lines at 1 on both axes denote manufacturer set cutoff for the Roche and Ortho NC assays.

All 200 pre-COVID-19 specimens collected in 2019 tested nonreactive on S and NC assays on both Roche and Ortho platforms. Testing of specimens captured from March and May 2021 demonstrated overall agreement of 96.4% (3,796/3,903; see Table 1 for qualitative results). The Ortho S and both NC assays had broad dynamic ranges spanning the observed reactivity values in the tested specimens (Fig. 2). The dynamic range of the Roche S assay is 0.4 to 250.0 Units/mL (U/mL; Fig. 2A) and a moderate proportion of reactive samples were at the upper limit of quantitation.

**TABLE 1:** Agreement of Roche and Ortho platforms for all S/NC reactivity classifications

	Roche			
Ortho	S-/NC-	S-/NC+	S+/NC-	S+/NC+
S-/NC-	1332	4	71	0
S-/NC+	7	6	0	0
S+/NC-	28	0	1398	2
S+/NC+	0	4	18	1033

**FIG 3:**
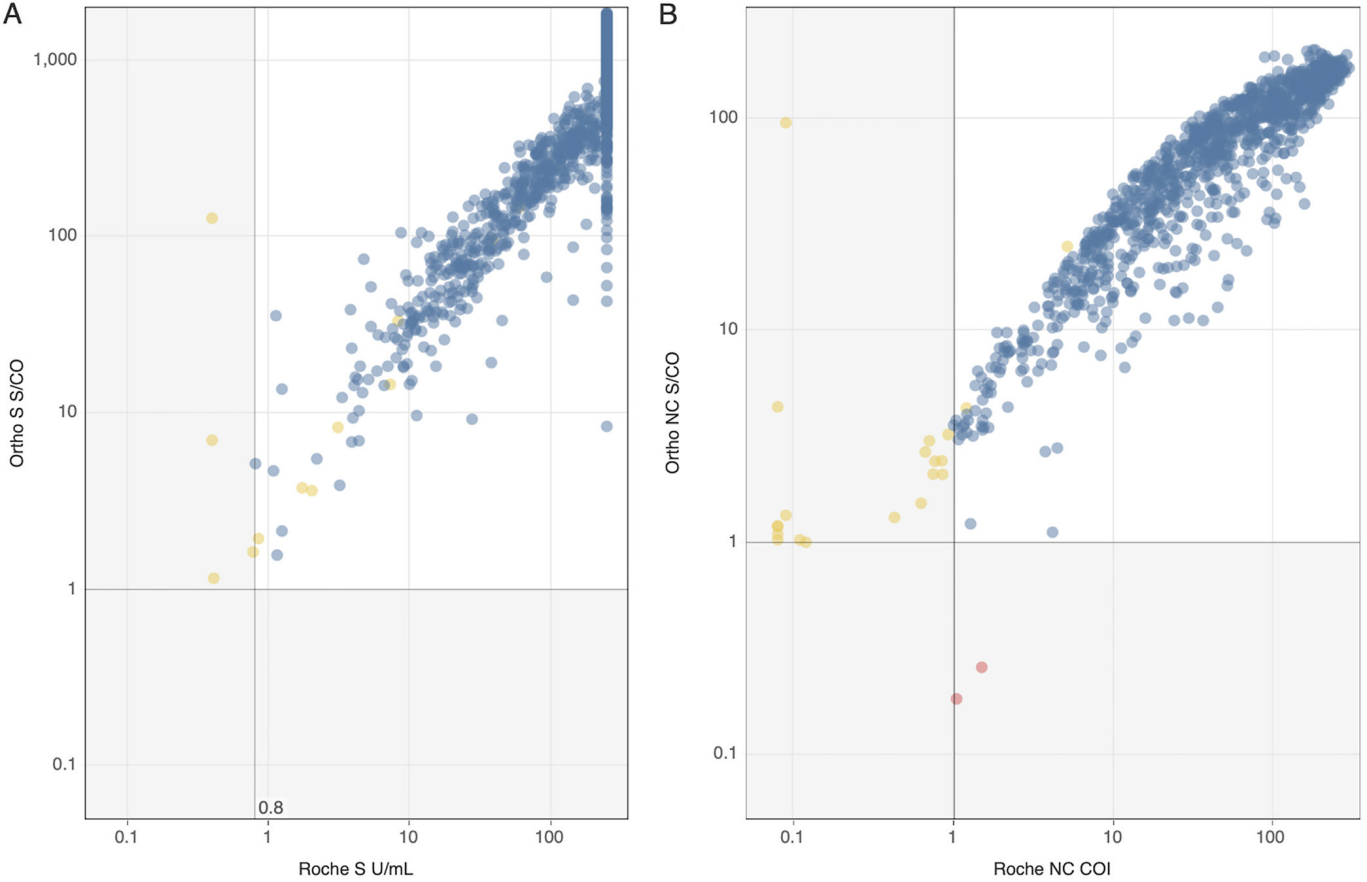
Numeric output of specimens tested S+/NC+ on either or both platforms for detection of seroreactivity resulting from prior SARS-CoV-2 infection (*n* = 1057). (A) Numeric outputs of the two S assays. Gray lines at 0.8 and 1 denote manufacturers’ cutoffs for the Roche and Ortho S assays, respectively. (B) Numeric outputs of the two NC assays. Gray lines at 1 on both axes denote manufacturers’ cutoffs for the respective assays. Blue marks denote S+/NC+ reactive samples on both platforms, yellow marks S+/NC+ on Ortho only, and red marks S+/NC+ on Roche only.

For detection of prior infection, defined as specimens with S and NC seroreactivity on either or both platforms, Roche classified 4 specimens as S-/NC+ and 18 as S+/NC- that tested S+/NC+ on the Ortho assays, while Ortho only classified 2 specimens as S+/NC- that tested S+/NC+ on Roche (Table 1). Eight of the 18 (44%) Ortho S+/NC+ specimens classified by Roche as S+/NC- showed gray zone reactivity below the manufacturer’s cutoff (COI >0.5 and <1.0) on the Roche NC assay; both Ortho S+/NC- specimens classified by Roche as S+/NC+ tested low-level reactive (COI >1.0 and <5.0) on the Roche NC assay (Fig. 3).

**FIG 4:**
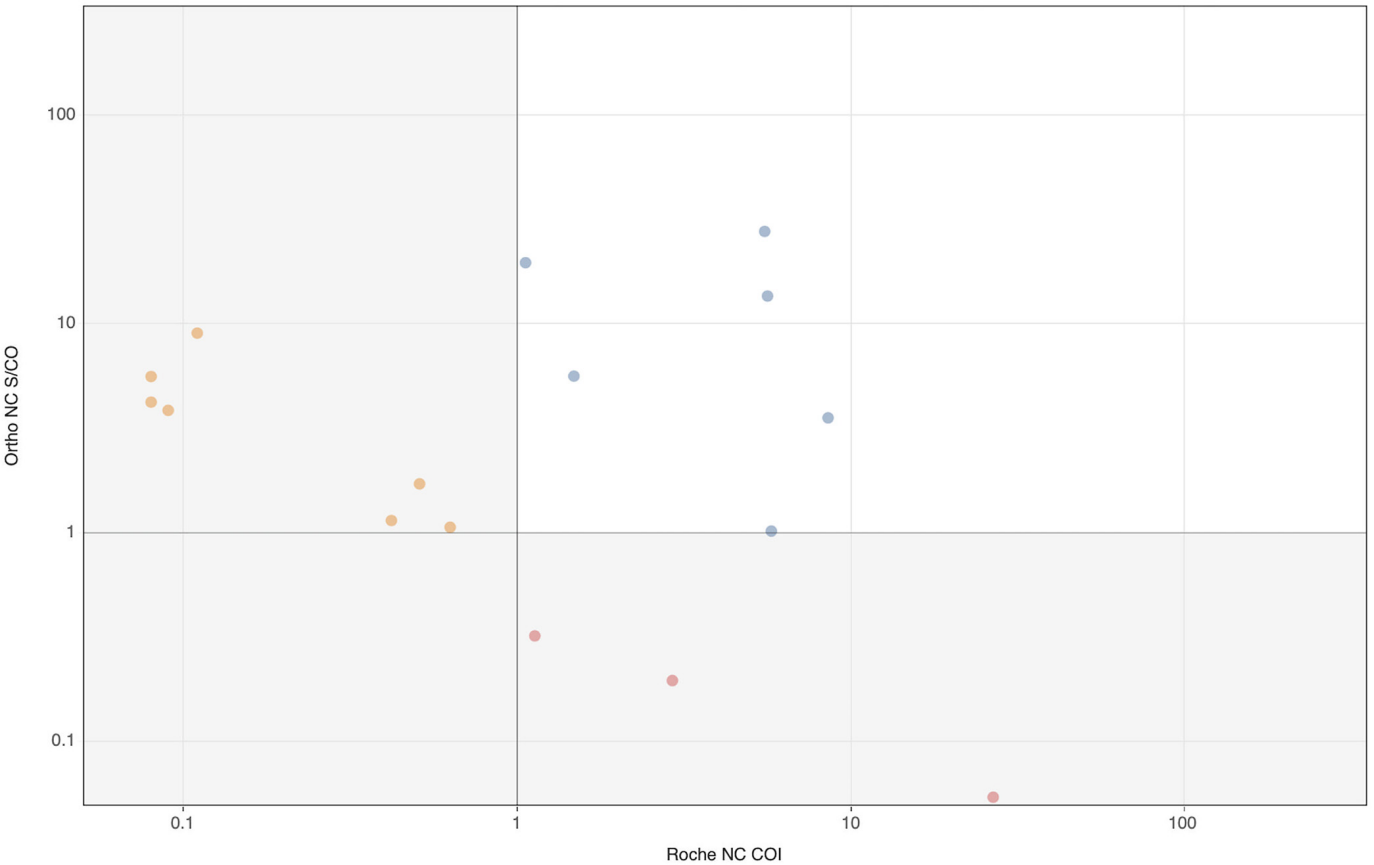
Roche and Ortho NC assay numeric outputs for specimens classified as S- on both platforms and NC+ on either or both platforms representing differential detection of NC-only reactive samples (*n* = 17). Blue, yellow, and red symbols denote specimens in agreement between the two platforms, NC- on the Roche platform, and NC- on the Ortho platform, respectively.

NC-only reactive specimens are relatively rare and could result from divergent serologic responses to infection or from early infections where NC antibodies were detected sooner than S antibodies (17). Among 17 specimens that were S-/NC+ on either platform, 6 (28%) were S-/NC+ on both platforms (Table 1). Ortho detected 7 NC-only reactive specimens which were classified as S-/NC- on Roche’s NC assay, whereas Roche detected 4 NC-only reactive specimens which were classified as S-/NC- on Ortho’s NC assay. As shown in Fig. 4, NC signal intensities of NC discordant specimens were markedly lower (mean S/CO 5.0 for Ortho NC and mean COI 3.5 for Roche NC) than the reactivity levels observed in concordant S+/NC+ specimens (mean S/CO 21.2 for Ortho NC and mean COI 19.9 for Roche NC assays, respectively; Fig. 3B).

**FIG 5:**
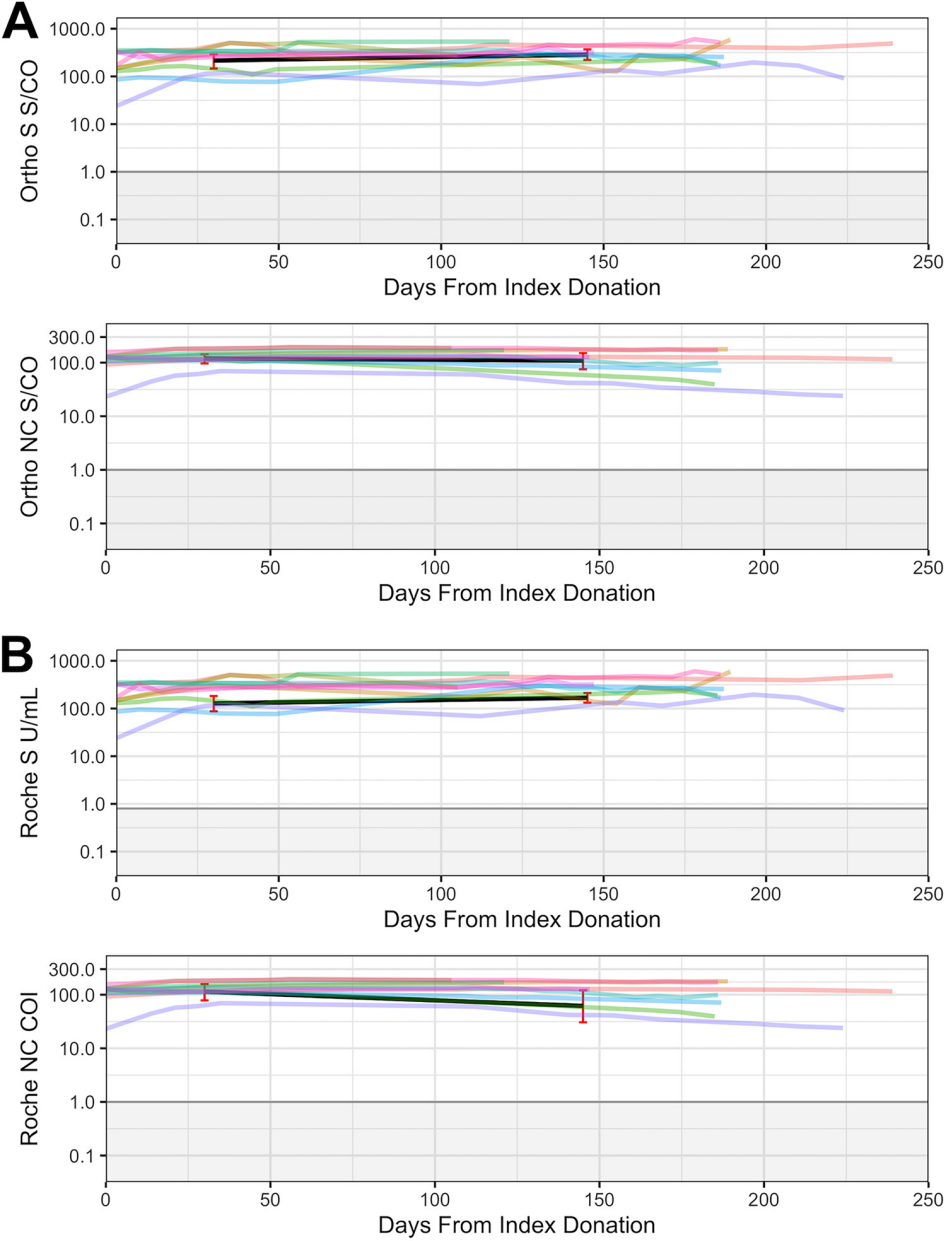
Testing results of longitudinal CCP donor specimens on Ortho and Roche assays/platforms (*n* = 133). (A) Ortho assays/platform results, (B) Roche assays/platform results. Black lines represent weighted geometric means for 0–60 and 100+ time periods for each assay. 95% confidence intervals, calculated using a bootstrapping method, are shown in red for each assay’s weighted geometric mean at each time period.

All longitudinal specimens tested were reactive at all time points on both Roche and Ortho platforms (Fig. 5). Weighted geometric means were calculated for all assays based on results for samples collected at 0 to 60 and 100+ days following first (index) CCP donations (Table S1). For the Ortho S assay, the means are 215.55 S/CO (95% CI:146.2 to 289.4) and 286.63 S/CO (95% CI:221.9 to 370.2), while for the Roche S assay the means are 127.99 U/mL (87.2 to 182.4) and 168.44 U/mL (132.2 to 211.4), indicating modestly increasing levels of seroreactivity over time. For the Ortho NC assay, the means are 119.6 S/CO (96.3 to 142.9) and 109.23 S/CO (75.1 to 150.6), while for Roche NC the means are 114.28 COI (78.3 to 157.6) and 61.77 COI (30.5 to 120.5), demonstrating stable anti-NC reactivity over time for the Ortho NC assay and modestly declining reactivity on the Roche NC assay.

## DISCUSSION

The NBDS study-wide spike antibody seroprevalence increased from 49.2% in March 2021 to 83.3% in May 2021 (14), which made the original testing algorithm of the NBDS program, based on reflex testing Ortho S-reactive specimens with the Roche NC assay, impractical due to the high volume of reflex Roche NC testing needed. While reflex testing could distinguish between vaccine-induced and infection-induced seroreactivity, increased vaccination uptake resulted in overwhelming rates of spike-reactivity which made it impractical. Reflex testing, in the context of our study, was more labor-intensive than parallel testing; requiring more steps to complete the testing algorithm, i.e., identification of samples to reflex test by interpreting the initial testing results, pulling and thawing of samples for reflex testing, having to code for separate data pipelines for each manufacturer and assay, etc. Consequently, in an effort to improve turnaround time for testing, the study shifted in the summer of 2021 to parallel S and NC testing on the Ortho platform, using the then recently FDA Emergency Use Authorized Ortho NC Total Ig assay. To validate the use of the Ortho NC assay prior to that change, we examined the assay’s performance relative to the Roche NC assay on preselected specimen sets reflecting all reactivity patterns. To better understand possible downstream implications of adopting parallel testing algorithms, we also performed comparative assessment of S and NC testing on the two platforms on study tested specimens from two monthly sampling periods.

In the preselected antibody discordant groups, numeric output on specimens classified differently by the two assays show some linearity in the gray zone signal intensities below both assay cutoffs (S/CO >0.5 and <1.0) (Fig. 1B and C). This suggests that lower cutoffs could be considered, although further research is needed to define these alternative cutoffs. A recent analysis of UK serosurvey samples recommended a threshold of 0.47 for the detection of vaccine breakthrough infections using the Roche NC assay (11, 18). As commercial manufacturers and FDA tend to maximize specificity when setting assay thresholds, sensitivity may be compromised to achieve optimal specificity; thus, specimens with gray zone reactivity likely represent infections. For serosurveillance, the optimal balance of sensitivity and specificity is different to the optimal balance for clinical or diagnostic applications, i.e., the benefits of increased sensitivity may outweigh the disadvantages of reduced specificity. This is especially true in situations where prevalence is high.

In our assessment of parallel testing on the two platforms, we have shown comparable performance between the Roche and Ortho S and NC assays, which supports application of parallel testing using either platform in serosurveillance. Both platforms demonstrated 100% specificity as well as 96.4% overall agreement. These findings demonstrate that seroprevalence studies using the Roche S and NC Total Ig assays, such as studies performed in the United States., Canada, and the U.K. (19, 20), are likely comparable to our NBDS study based on the Ortho S and NC Total Ig assays (14).

Discrepancies between Roche and Ortho S/NC antibody classifications were infrequent and could be attributable to differences in manufacturer established cutoffs and/or the antigens targeted by the respective assays. As most samples with discordant classifications had near-cutoff gray zone reactivity on the assay with negative results, adjusting assay cutoffs may increase concordance. Although the Ortho NC assay appears to be slightly more sensitive than the Roche NC assay (~2% increased detection of NC antibodies), the overall impact on seroprevalence studies would be minimal. While both NC assays had specificity of 100% based on our testing of prepandemic samples, we cannot exclude that increased detection of the Ortho NC assay represents false reactivity. Adjusting assay cutoffs to optimize performance for serosurveillance requires consideration of contextual factors such as prevalence in the population of interest. For example, in populations with relatively lower levels of NC seroreactivity and high rates of vaccination where a substantial proportion of infections occur after vaccination, lowering an NC assay cutoff may result in small increases to the assay’s detection of past infections (18, 20).

The ability to durably detect antibody responses from past infections long after symptom resolution is critical for accurate estimation of cumulative vaccination rates and incidence of infections. The Roche and Ortho platforms showed comparable performance in durability of S and NC antibody detection on longitudinal specimens and are thus both appropriate for use in serosurveillance applications (Table S1; Fig. 5).

Limitations in this study include a limited number of parallel tested samples and the lack of vaccine breakthrough samples to support formal analysis of gray zone reactivity to establish sub-cutoff thresholds for NC assays for detection of breakthrough infections.

In conclusion, performance of Ortho NC and Roche NC assays for detection of prior infection, as well as parallel S/NC testing on both platforms, were shown to be comparable, which supports the implementation of parallel testing using either platform in serosurveillance efforts requiring high volume testing and timeliness of result reporting. In the context of the NBDS program, though operational data were not collected, switching to parallel testing on a single platform resulted in a noticeable improvement to turnaround time for testing as the process became more operationally streamlined (i.e., more efficient reagent management, less equipment maintenance, less technician training, etc.). Therefore, given comparable performance of the platforms, considerations such as platform availability, cost, and operational feasibility should inform decisions on which platform to use for serosurveillance.

## MATERIALS AND METHODS

### Sample selection.

Plasma specimens were obtained from multiple sources (Table 2). Blood donor specimens derived from fresh frozen plasma components collected before the pandemic in 2019 were included to assess specificity. Donor specimens included in the NBDS Study were selected from Versiti Blood Center (VST), Gulf Coast Blood Center (GCBC), and The Blood Center (TBC, based in New Orleans) sites, where universal NC testing with the Roche NC assay was being performed to screen all blood donations. Because these blood collection organizations were participating in the NBDS, all blood donation specimens included in the study were also tested using the Ortho S assay. For the validation of Ortho NC assay, we selected reactive specimens with all possible Ab-reactivity combinations (Ortho S+/Roche NC+, Ortho S+/Roche NC-, and Ortho S-/Roche NC+) to assess the performance of the Ortho NC assay relative to the Roche NC assay and examined assay specificity using specimens collected in 2019 prior to the start of the pandemic (Table 2).

**TABLE 2:** Specimen groups and the number of specimens per group included in validation of the Ortho NC assay and evaluation of parallel S and NC testing on Roche and Ortho platforms for serosurveillance

Group	Description	no. of specimens
Specificity	Prepandemic blood donor specimens collected prior to 2020 and demonstrated to be anti-SARS-CoV-2 negative by RVP neutralization testing	200
Validation of Ortho NC to replace Roche NC	Preselected Ortho S+/Roche NC- specimens from before vaccines were widely available (July-December 2020)	367
	Preselected Ortho S-/Roche NC+ specimens collected between November 2020 and March 2021	37
	Preselected antigen concordant reactive (Ortho S+/Roche NC+) specimens from before vaccines were widely available (September-December 2020)	190
Evaluation of Parallel S/NC Testing on Ortho and Roche Platforms	Specimens captured through MASS-BD sampling from The Blood Center (New Orleans) for March (*n* = 1982) and May 2021 (*n* = 1921)	3903
Durability of Antibody Detection	Longitudinal plasma specimens from 10 convalescent plasma donors	133

To compare Roche S/NC and Ortho S/NC assay performance on vaccinated and unvaccinated blood donor specimens, specimens from routine testing by TBC for March and May 2021 were tested on all four assays (April 2021 specimens were unavailable for testing). Lastly, 7-20 longitudinal specimens from 10 consenting COVID-19 Convalescent Plasma (CCP) donors followed for 105 to 239 days (mean: 177 days) were tested on all four assays to examine durability of antibody detection.

### Serology testing.

All specimens were tested for anti-S and anti-NC antibodies on both Ortho (Ortho Clinical Diagnostics VITROS Anti-SARS-CoV-2 Total Antibody Test, Vitros 3600 & Vitros 7600, Raritan, NJ) and Roche (Roche Elecsys Anti-SARS-CoV-2 antibody test, Roche cobas e 411, Basel, Switzerland) platforms. Parallel Ortho S/NC testing was performed at VRI in San Francisco, California, and Creative Testing Solutions (CTS) in Tempe, Arizona. Roche S and NC testing was performed at The Blood Center in New Orleans, Louisiana, and at the Department of Pathology and Laboratory Medicine at UC Davis. All testing, quality control, and calibration were performed per manufacturer’s instructions for use.

### Statistical analyses.

We compared assay performance, including agreement using groups of preselected specimens from routine NBDS sampling and specificity using prepandemic samples. To evaluate the performance of S and NC assays on both Ortho and Roche platforms for use in serosurveillance, agreement (e.g., percentage of Roche S+/NC+ that were Ortho S+/NC+) was evaluated and a correlation test for the Roche NC and Ortho NC was performed using Spearman’s correlation coefficient. To examine durability of antibody detection, the weighted geometric mean of each assay’s numeric output on the longitudinal CCP donor specimens was calculated for two date ranges:1 to 60 and 100+ days from index donation. Weighting was done based on the inverse of the number of donations the donor had provided within the time periods so that donors with variable numbers of specimens in each time range would have the same weight. To compute 95% confidence intervals on geometric means, we performed 10,000 iterations of donor-level bootstrapping (i.e., resampling donors with replacement) to reflect intersubject variability given that multiple measurements from the same donor were expected to be correlated. All statistical analyses were performed using R version 3.5.0 (15). All data and R scripts for the study is available for download from Dryad (16).

### Institutional review board statement.

All blood donors consented to use of deidentified, residual specimens for further research purposes. Consistent with the policies and guidance of the University of California–San Francisco Institutional Review Board, Vitalant Research Institute self-certified the use of deidentified donations in this study as not meeting the criteria for human subjects research. Centers for Disease Control and Prevention (CDC) investigators reviewed and relied on this de-termination as consistent with applicable federal law and CDC policy (45 C.F.R. part 46, 21 C.F.R. part 56; 42 U.S.C. § 241[d]; 5 U.S.C. § 552a; 44 U.S.C. § 3501).
