# Supplementary material for: Evaluation of Ortho VITROS and Roche Elecsys S and NC Immunoassays for SARS-CoV-2 Serosurveillance Applications
Source: Microbiol Spectr. 2023 Jun 22;11(4):e03234-22. doi: 10.1128/spectrum.03234-22 (PMC10434072; doi:10.1128/spectrum.03234-22)
Supplement: Supplemental file 1 — Table S1. Download spectrum.03234-22-s0001.pdf, PDF file, 0.05 MB [file spectrum.03234-22-s0001.pdf]

Supplementary Materials

Table S1. Weighted geometric means for each assay and time period. 95% CI was calculated using a bootstrapping method.

Assay	Time Period	
	0-60 Days	100+ Days
Ortho S	215.6 (146.2-289.4)	286.6 (221.9-370.2)
Ortho NC	119.6 (96.3-142.9)	109.2 (75.1-150.6)
Roche S	128.0 (87.2-182.4)	168.4 (132.2-211.4)
Roche NC	114.3 (78.3-157.6)	61.8 (30.5-120.5)
